# Specific Compounds Derived from Traditional Chinese Medicine Ameliorate Lipid-Induced Contractile Dysfunction in Cardiomyocytes

**DOI:** 10.3390/ijms25158131

**Published:** 2024-07-25

**Authors:** Fang Wang, Dietbert Neumann, Dimitris Kapsokalyvas, Martijn F. Hoes, Francesco Schianchi, Jan F. C. Glatz, Miranda Nabben, Joost J. F. P. Luiken

**Affiliations:** 1 Department of Genetics & Cell Biology, Maastricht University, 6229 ER Maastricht, The Netherlands; f.wang@maastrichtuniversity.nl (F.W.); d.kapsokalyvas@maastrichtuniversity.nl (D.K.); schianchif90@hotmail.it (F.S.); j.luiken@maastrichtuniversity.nl (J.J.F.P.L.); 2 Cardiovascular Research Institute Maastricht, Faculty of Health, Medicine and Life Science, Maastricht University, 6229 ER Maastricht, The Netherlands; d.neumann@maastrichtuniversity.nl (D.N.); martijn.hoes@maastrichtuniversity.nl (M.F.H.); 3 Department of Pathology, Maastricht University Medical Center+, 6229 HX Maastricht, The Netherlands; 4 Interdisciplinary Centre for Clinical Research IZKF, University Hospital RWTH Aachen, 52074 Aachen, Germany; 5 Department of Cardiology, Maastricht University, 6229 ER Maastricht, The Netherlands; 6 Department of Clinical Genetics, Maastricht University Medical Center+, 6229 HX Maastricht, The Netherlands; glatz@maastrichtuniversity.nl

**Keywords:** lipotoxic cardiomyopathy, insulin resistance, endosomal CD36 and GLUT4, contractile function, adult rat cardiomyocytes

## Abstract

Chronic lipid overconsumption, associated with the Western diet, causes excessive cardiac lipid accumulation, insulin resistance, and contractile dysfunction, altogether termed lipotoxic cardiomyopathy (LCM). Existing treatments for LCM are limited. Traditional Chinese Medicine (TCM) has been shown as beneficial in diabetes and its complications. The following compounds—Resveratrol, Quercetin, Berberine, Baicalein, and Isorhamnetin—derived from TCM and often used to treat type 2 diabetes. However, virtually nothing is known about their effects in the lipid-overexposed heart. Lipid-induced insulin resistance was generated in HL-1 cardiomyocytes and adult rat cardiomyocytes by 24 h exposure to high palmitate. Upon simultaneous treatment with each of the TCM compounds, we measured myocellular lipid accumulation, insulin-stimulated fatty acid and glucose uptake, phosphorylation levels of AKT and ERK1/2, plasma membrane appearance of GLUT4 and CD36, and expression of oxidative stress-/inflammation-related genes and contractility. In lipid-overloaded cardiomyocytes, all the selected TCM compounds prevented lipid accumulation. These compounds also preserved insulin-stimulated CD36 and GLUT4 translocation and insulin-stimulated glucose uptake in an Akt-independent manner. Moreover, all the TCM compounds prevented and restored lipid-induced contractile dysfunction. Finally, some (not all) of the TCM compounds inhibited oxidative stress-related *SIRT3* expression, and others reduced inflammatory *TNFα* expression. Their ability to restore CD36 trafficking makes all these TCM compounds attractive natural supplements for LCM treatment.

## 1. Introduction

Cardiovascular diseases remain a leading cause of morbidity and mortality worldwide. The rising prevalence of obesity and overweight adds to this health challenge, prompting continuous exploration of nuanced pathological entities [1]. The myocardium is traditionally viewed as a metabolic omnivore capable of utilizing various substrates for energy production so as to secure a proper energy provision during divergent conditions [2]. However, chronic long-term overexposure of lipids might cause harmful effects to the heart [3]. Lipotoxicity, characterized by the accumulation of lipids, particularly fatty acids, within non-adipose tissues, has assumed prominence as a key player in the intricate pathogenesis of lipotoxic cardiomyopathy [4]. The pathophysiology of lipid-induced cardiomyopathy (LCM) is still under investigation, although, in recent decades, a wide variety of mechanisms have been suggested to be involved. The main metabolic abnormalities contributing to cardiac dysfunction are disorders of glucose and lipid metabolism, mainly the switch in cardiac metabolism away from glucose towards increased fatty acids metabolism, and the development of insulin resistance [5]. These latter abnormalities are known to cause changes in energy substrate metabolism and cardiac lipotoxicity and further lead to inflammation, oxidative stress, and cardiac contractile dysfunction [3,6]. 

Cardiac metabolic energy is produced predominantly from the oxidation of long-chain fatty acids (LCFA) and glucose. Under normal conditions, LCFA contribute to total energy production by about 50–60% and glucose by about 30–40%, while the remaining 10% is covered primarily by lactate, ketone bodies, and amino acids [2]. In LCM, there is an excessive supply of LCFA to the heart due to chronic lipid overconsumption (as part of a Western diet). This results in excessive uptake of LCFA into the myocardial cells. While most LCFA are stored as inert lipid droplets, the surplus of LCFA that is not used for energy production gives rise to increased levels of bioactive lipid metabolites, such as diacylglycerol (DAG) and ceramides, which eventually cause insulin resistance and impaired contractile function [7]. Taken together, it appears that lipid overconsumption is often at the start of the above-mentioned maladaptive cascade leading to LCM. Indeed, interventions aimed at normalizing cardiac lipid metabolism have been found to be effective therapeutic approaches for LCM [8,9]. 

In recent years, it has been established that the lipid transporter CD36 and glucose transporter GLUT4 which facilitate LCFA and glucose uptake into cardiomyocytes, respectively, are primarily responsible for the proper regulation of myocardial metabolism [10]. Both transporters are located at the sarcolemma (cell surface) but are also, for a substantial part, stored in intracellular membrane compartments (endosomes). In a healthy heart, hormonal and mechanical stimuli induce the (reversible) translocation of both transporters to the sarcolemma via vesicular trafficking. Thus, in a normal situation, the vesicle-mediated recycling of CD36 and GLUT4 between endosomes and the sarcolemma appears to be closely adjusted to the cellular energy needs, avoiding too-low uptake rates (risk of energy deficit) and too-high uptake rates (risk of lipotoxicity or glucotoxicity). As a result, CD36 and GLUT4 serve as a dynamic gatekeepers of myocardial lipid metabolism [11]. 

During lipid overload conditions in the heart, total cellular CD36 (protein) expression does not increase, yet there is an increase in its content at the sarcolemma. This increased cell surface CD36 abundance is accompanied by a decrease in its presence within the endosomes and, more specifically, the endosomal sub-compartment that is responsive to insulin [12,13,14]. The underlying mechanism is increased translocation of CD36 under conditions of high lipid supply. The consequent chronic increase in fatty acid uptake will then cause accumulation of diacylglycerol/ceramides and inhibition of insulin signaling and of insulin-stimulated GLUT4 translocation. Taken together, in the lipid-overloaded heart, both insulin-stimulated CD36 translocation and insulin-stimulated GLUT4 translocation will get lost, but the underlying mechanisms are different, given that basal CD36 translocation, but not that of GLUT4, is increased [10]. Additionally, both maladaptive events are separated in time, with the loss of insulin-stimulated CD36 translocation being an early event and the loss of insulin-stimulated GLUT4 translocation following later with a considerable time gap [13]. Thereafter, oxidative stress, inflammation, and reduced contractility will also develop due to the lipotoxic conditions.

Although LCM has become a serious worldwide public health hazard, so far, there are no effective and safe drugs to treat it. Traditional Chinese Medicine (TCM) is a complex formula composed of mixed medicinal extracts from Chinese herbs and animals. TCM has been implicated in practical use in China for thousands of years, and its practitioners have accumulated substantial front-line experience in treating various diseases with effectiveness and few side effects, particularly for diabetes [15,16,17]. Studies on TCM have demonstrated that their active ingredients show beneficial effects on insulin resistance and lipid metabolism both in vitro and in vivo [18]. In recent years, great interest has been focused on TCM, producing a substantial amount of research data [15]. However, few data are available on the effects of specific TCM compounds on isolated cardiomyocytes challenged by lipid overload.

The TCM compounds Resveratrol, Quercetin, Berberine, Baicalein, and Isorhamnetin were selected from the TCMSP database (https://old.tcmsp-e.com/tcmsp.php, accessed on 8 November 2023). All five compounds exhibit strong antioxidant and anti-inflammatory properties, showing potential in combating diabetes and cardiovascular diseases. Below, we summarize the information for each compound that led to its selection. Resveratrol is a stilbenoid attenuating high-fat-diet-induced insulin resistance by influencing skeletal muscle lipid transport and subsarcolemmal mitochondrial β oxidation [19,20] and inhibiting the expression of CD36 in high-fat-diet mice [21]. The flavonoid Quercetin has been used in the clinic for treating type 2 diabetes, and Quercetin intake was inversely related to the prevalence of T2DM in the Chinese population [22]. Quercetin also alleviates high-fat-diet-induced lipid accumulation by reducing CD36 expression in the liver and skeletal muscle [23,24]. Berberine is a quaternary ammonium isoquinoline alkaloid showing therapeutic effects in T2DM, improving insulin resistance. Berberine was also found to increase skeletal muscle GLUT4 protein expression in T2DM rats and decrease CD36 expression in adipocyte and liver [25,26,27]. The flavonoid Baicalein improves glucose metabolism in insulin-resistant HepG2 cells and adipocytes via increasing pAKT and GLUT4 translocation [28,29]. Although there is no evidence showing that Baicalein inhibits the expression of CD36, one study found that Baicalein suppressed oxLDL uptake through competitively inhibiting the binding of CD36 to the epitope structure of oxLDL [30]. Isorhamnetin is a flavonoid showing anti-inflammatory and anti-obesity effects [31]. It ameliorates insulin resistance, oxidative stress, and inflammation in T2DM mice in skeletal muscle [32] and also inhibits CD36 expression in adipocytes [33].

Most of the referenced studies investigating these compounds showed that they were effective in treating diabetes, but there was more focus on liver or skeletal muscle, rather than the heart. Moreover, these compounds have never been investigated in the context of LCM. In the present study, we investigated whether these compounds had possible beneficial effects on the onset of cardiac lipid accumulation, insulin resistance, oxidative stress, inflammation status and contractile force under cardiac lipid overload conditions *in vitro*, i.e., in high palmitate-treated cardiomyocytes. Additionally, we aimed to gain more insight into the main underlying mechanism by which these compounds operate, focusing on the lipid metabolic pathway. We show that each of these compounds exhibits therapeutic potential by protecting cardiomyocytes from lipid overload-induced damage, specifically, decreasing lipid accumulation, improving insulin sensitivity, as well as preventing and reversing contractile dysfunction.

## 2. Results

### 2.1. TCM Compounds Can Prevent Lipid Accumulation and Restore Intracellular Storage of CD36 Transporter Vesicles in Lipid-Overloaded Adult Rat Cardiomyocytes 

First, the impact of each individual TCM compound on cell viability was assessed by using the SRB test. When added at higher concentrations, each of the studied TCM compounds appeared to show a significant reduction in cell viability, whereas when applied at the lowest concentration viability, it was unaffected (Supplementary Figure S1A–E). Therefore, in subsequent experiments, all five TCM compounds were added at the lowest of the tested concentrations.

**Figure 1:**
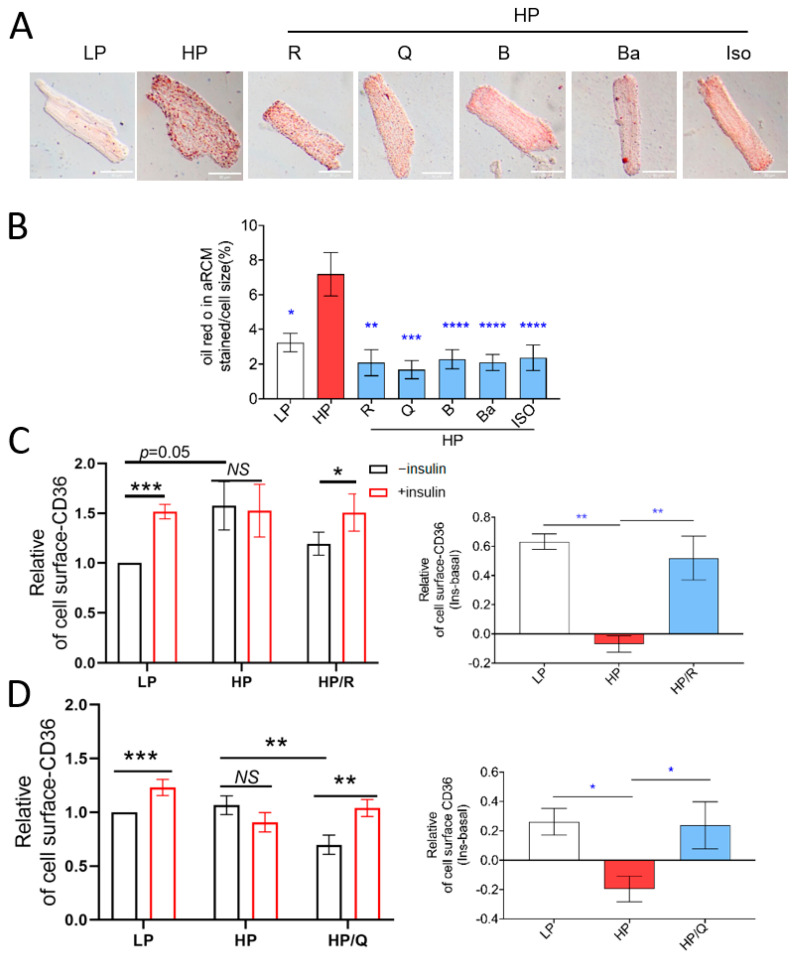

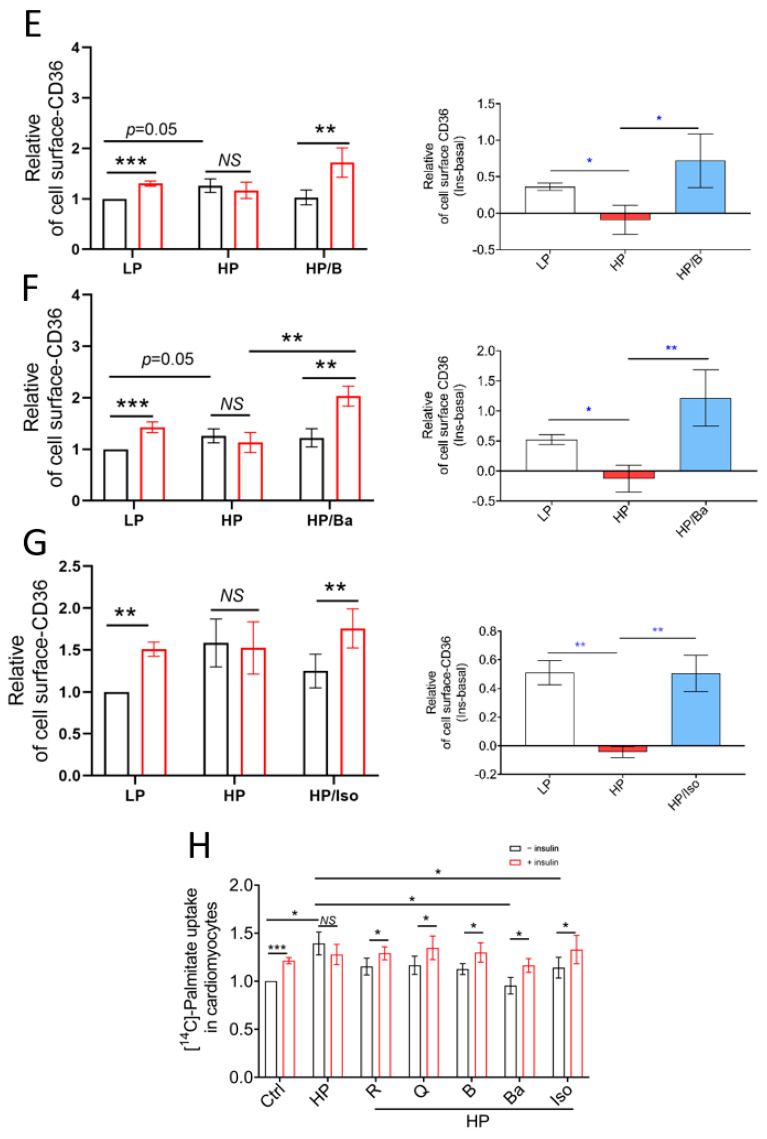
TCM compounds can prevent lipid accumulation and restore intracellular storage of CD36 transporter vesicles in lipid-overloaded adult rat cardiomyocytes. In panels (**A**–**H**), cells were incubated for 24 h under different conditions: low palmitate (LP, basal condition for aRCMs; palmitate/BSA ratio 0.3:1); high palmitate (HP, palmitate/BSA ratio 3:1); or HP supplemented with Resveratrol (R), Quercetin (Q), Berberine (B), Baicalein (Ba), or Isorhamnetin (Iso). (**A**,**B**): Lipid droplet content was assessed via Oil red O staining. Representative Oil red O images of aRCMs are displayed (scale bar = 30 μm) (**A**). Images were quantified using Image J-Win-64, (*n* = 5) (**B**). (**C**–**G**): Assessment of cell-surface CD36 in aRCMs. After 24 h, cells were short-term (30 min) incubated without/with 100 nM insulin. Then, cells were subjected to the biotinylation assay. CD36 was assessed using Western blotting in both biotin immunoprecipitations and total cell lysates, followed by quantification. In the left panel, basal and insulin-stimulated cell surface CD36 levels were measured, while the right panel shows the delta insulin effect on cell surface CD36, R (*n* = 4), Q (*n* = 5), B (*n* = 4), Ba (*n* = 5), and Iso (*n* = 4). Representative Western blots of biotinylated CD36 and total CD36 are displayed in Supplementary Figure S3A–E, left panel. (**H**) [^14^C] Palmitate uptake in HL-1 cells (*n* = 6). White columns represent basal condition, red columns represent HP condition, blue columns represent prevention of TCM compounds. In panels (**C**–**G**, left) and H, white columns represent basal condition, red represent insulin stimulation condition. Data are presented as mean ± SEM. One-way ANOVA followed by Duncan’s post hoc tests (among the groups, i.e., different culturing conditions) was used for the comparison. *NS* represents non significant, * *p* < 0.05 was considered statically significant. (* *p* < 0.05, ** *p* < 0.01, *** *p* < 0.001, **** *p* < 0.0001).

In lipid-overloaded cardiomyocytes, CD36 translocation to the sarcolemma is known to contribute to an increase in fatty acid uptake and myocellular lipid accumulation [10]. Using Oil red O staining (Figure 1A,B), we confirmed that HP increased myocellular lipid accumulation (2.2-fold; Figure 1B). If administered in conjunction with HP incubation, all five selected compounds reduced myocellular lipid droplet levels (Figure 1A,B).

Given that maladaptive lipid accumulation is due to increased CD36 translocation and subsequently increased fatty acid uptake (see Introduction), we investigated the effects of all five compounds on cell-surface CD36 levels. As expected [34], culturing in HP medium induced CD36 translocation to the cell surface (increase in cell-surface CD36 vs. basal; Supplementary Figure S4 *n* = 22; *p* < 0.05). In the LP condition, short-term (30 min) insulin stimulation triggered cell-surface CD36 translocation (Figure 1C–G left panel; *p* < 0.05). In contrast, in the HP condition, insulin did not stimulate CD36 translocation (Figure 1C–G). Importantly, all five selected TCM compounds successfully preserved insulin-stimulated CD36 translocation during HP incubation. This preservation of insulin-stimulated CD36 translocation is only possible when CD36 internalization has been restored. We also measured fatty acid uptake following CD36 translocation. Indeed, HP culturing increased basal fatty acid uptake rates at the cost of insulin stimulation. Only Baicalein and Isorhamnetin significantly decreased basal fatty acid uptake. Yet, importantly, all five compounds preserved insulin-stimulated fatty acid uptake in line with their beneficial effects on insulin-stimulated CD36 translocation (Figure 1H). Altogether, in cardiomyocytes exposed to excess lipids, all the selected TCM compounds prevented myocellular lipid accumulation due to the restoration of CD36 trafficking.

### 2.2. TCM Compounds Partially Prevent Decreased Insulin Signaling in Lipid-Overexposed Cardiomyocytes, but Completely Preserve Insulin-Stimulated Glucose Uptake in Lipid-Overexposed Cardiomyocytes

**Figure 2:**
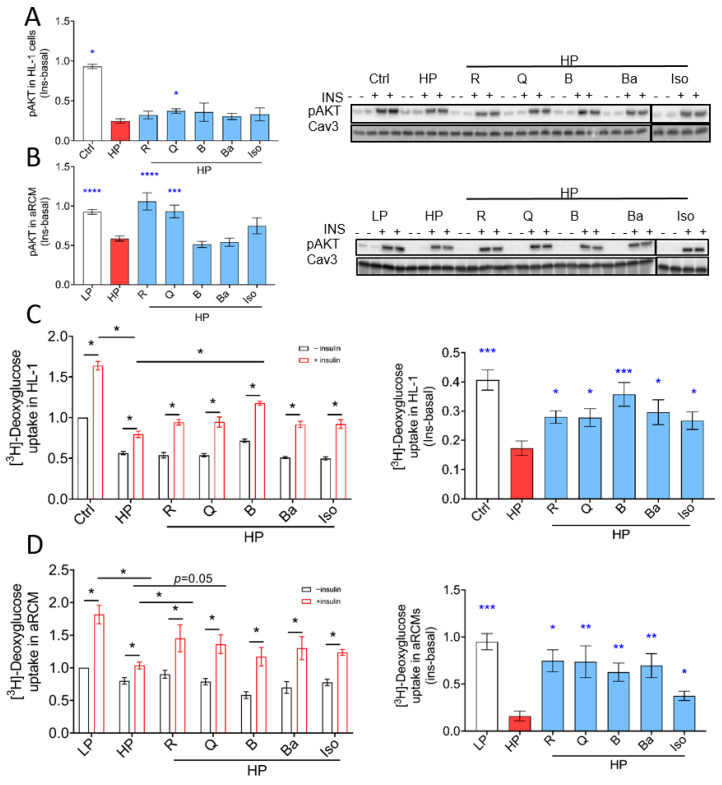

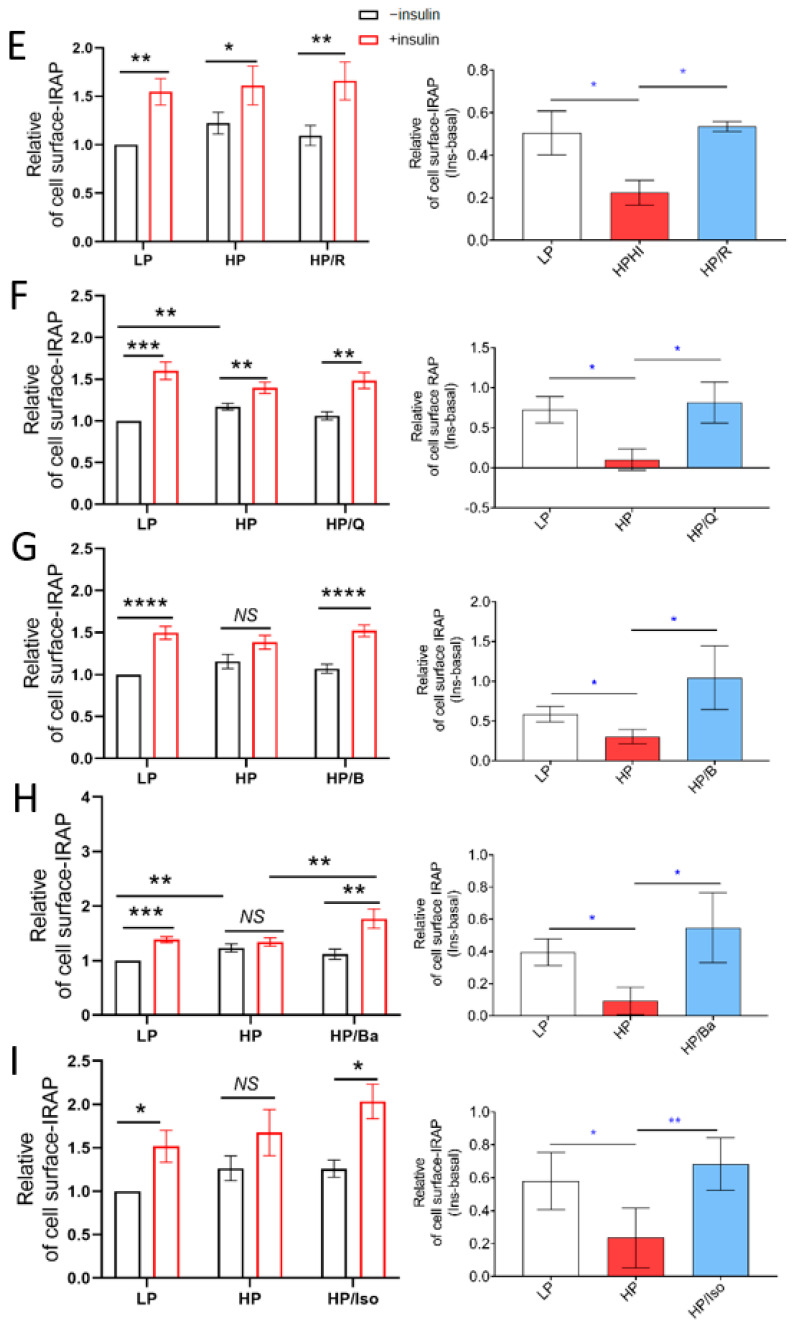

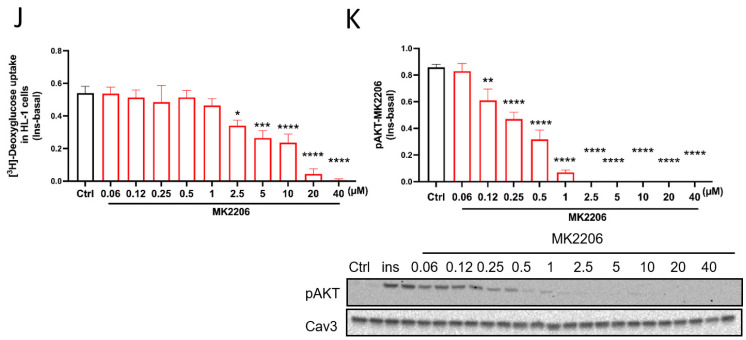
TCM compounds partially prevent decreased insulin signaling in lipid-overexposed cardiomyocytes but preserve insulin-stimulated glucose uptake in lipid-overexposed cardiomyocytes. In panels (**A**–**K**), cells were cultured for 24 h under various conditions, being ctrl (no palmitate; the basal condition for HL-1), HP medium (containing 500 μM palmitate and 100 nM insulin; HP condition for HL-1), low palmitate (LP, basal condition for aRCMs), high palmitate (HP, palmitate/BSA ratio 3:1), and HP supplemented with Resveratrol (R), Quercetin (Q), Berberine (B), Baicalein (Ba), or Isorhamnetin (Iso). After 24 h, cells were short-term (30 min) incubated without/with insulin (HL-1 cells: 200 nM insulin; aRCMs: 100 nM insulin). (**A**,**B**). Phosphorylation of AKT (p-AKT ser473) in HL-1 cells (*n* = 6) and aRCMs (*n* = 3) was detected by Western blotting. For quantitative comparison of p-AKT among the different conditions, these signals were normalized against the respective signal of caveolin-3 protein content (loading control). Representative Western blots of p-AKT and Cav-3 are displayed. (**C**,**D**). [^3^H] Deoxyglucose uptake in HL-1 cells (*n* = 6) and aRCMs (*n* = 6). (**E**–**I**). Assessment of the cell surface GLUT4 in aRCMs stimulated with LP medium, HP, or HP supplemented with R (**E**), Q (**F**), B (**G**), Ba (**H**), or Iso (**I**). In the left panel, basal and insulin-stimulated cell-surface GLUT4 levels were measured, while the right panel shows the delta insulin effect of cell-surface GLUT4, R (*n* = 4), Q (*n* = 5), B (*n* = 4), Ba (*n* = 5), and Iso (*n* = 4). Representative Western blot and quantification of insulin-regulated aminopeptidase (IRAP, which reflects GLUT4 translocation) are shown in biotin immunoprecipitation and total lysate fraction. (Supplementary Figure S3A–E, right panel). (**J**,**K**). [^3^H] Deoxyglucose uptake and phosphorylation of AKT (p-AKT ser473) in HL-1 cells treated with MK2206. HL-1 cells were cultured for 24 h under various conditions, being ctrl, HP, and HP supplemented with various concentrations of the pan-AKT inhibitor MK2206 (HP/MK2206) (**J**). [^3^H] Deoxyglucose uptake in HL-1 cells treated with MK2206. After 24 h, cells were short-term (30 min) incubated without/with insulin (HL-1 cells: 200 nM insulin). Then, cells were subjected to [^3^H] Deoxyglucose uptake assay (*n* = 4). (**K**). Phosphorylation of AKT (p-AKT ser473) in HL-1 cells treated with MK2206 was detected by Western blotting (*n* = 4). White columns represent basal condition, red columns represent HP condition, blue columns represent prevention of TCM compounds. In panels (**C**–**I**, left), white columns represent basal condition, red represent insulin stimulation condition. (**J**,**K**), white columns represent basal condition, red columns represent MK2206 inhibition condition. Bar values are means ± SEM. One-way ANOVA followed by Duncan’s post hoc tests (among the groups, i.e., different culturing conditions), or paired Student’s *t*-test (within groups, i.e., when analyzing short-term insulin effect) was used for the comparison (the comparisons were indicated either by a line or compared to Ctrl (**J**,**K**)). *NS* represents non significant, * *p* < 0.05 was considered statically significant. (* *p* < 0.05, ** *p* < 0.01, *** *p* < 0.001, **** *p* < 0.0001).

It is known that increased CD36-mediated fatty acid uptake and lipid accumulation precede the loss of insulin-stimulated glucose uptake and the associated development of insulin resistance [34]. We therefore investigated if the five selected TCM compounds would preserve insulin sensitivity in lipid-overexposed cardiomyocytes. For the evaluation of insulin signalling, the phosphorylation of AKT (pAKT Ser 473) was assessed. As expected, HP exposure caused a loss of insulin-stimulated phosphorylation of AKT in both HL-1 cells (Figure 2A) and aRCM (Figure 2B). In HL1 cells, only Quercetin had a significant, but modest, beneficial effect, and in aRCMs, Resveratrol and Quercetin showed the ability to prevent the HP-induced loss of insulin-stimulated AKT phosphorylation.

Inhibition of insulin-stimulated glucose uptake is another well-described maladaptive action of myocellular lipid overload and directly downstream of impairment of the classical insulin signaling pathway involving AKT phosphorylation. HP incubation indeed inhibited insulin-stimulated glucose uptake (Figure 2C,D; *p* < 0.05), whereas all five compounds significantly restored the insulin effect in both HL-1 and aRCM (Figure 2C,D; *p* < 0.05). As was done for CD36 translocation (Figure 1C–G), we also assessed insulin-stimulated GLUT4 cell surface abundance for all five compounds (Figure 2E–I). Accordingly, Resveratrol, Quercetin, Berberine, Baicalein, and Isorhamnetin also prevented the HP-induced loss of insulin-stimulated GLUT4 translocation.

When combining the findings on insulin signaling and insulin-stimulated GLUT4 translocation/glucose uptake, there is an apparent discrepancy between the lack of preservation of insulin signaling by the selected TCM compounds and the complete preservation of insulin-stimulated glucose uptake/GLUT4 translocation in lipid-overloaded cardiomyocytes by all these compounds. This discrepancy made us hypothesize that a preservation of insulin-stimulated glucose uptake occurs, at least to a certain extent, independently of insulin signaling. To test this hypothesis, we applied a pan-Akt inhibitor (MK2206). The results show that insulin-stimulated Ser473 phosphorylation of Akt can be markedly inhibited by MK2206 at 1 μM without affecting insulin-stimulated glucose uptake (Figure 2J,K), indicating that it is difficult to make a close association between the activation of Akt and the insulin-stimulated translocation of GLUT4. These findings provide a more nuanced understanding of how the selected TCM compounds completely preserved insulin-stimulated glucose uptake while only partially preserving insulin signaling.

### 2.3. TCM Compounds Can Increase Antioxidant Levels and Prevent Increased Expression of Inflammatory Markers in Lipid-Overexposed Cardiomyocytes

**Figure 3:**
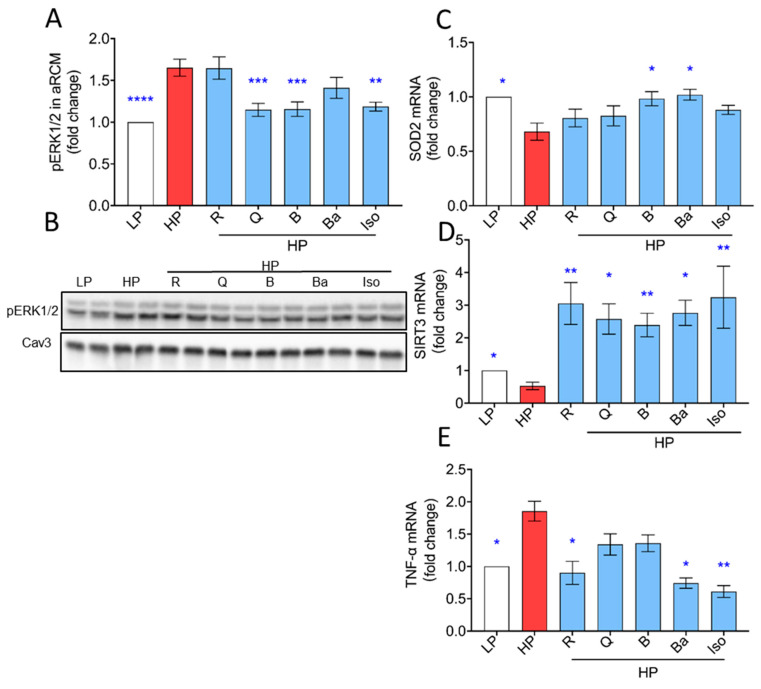
TCM compounds increase expression of antioxidant genes and prevent increased expression of inflammatory markers in lipid-overexposed cardiomyocytes. In (**A**–**E**) panels, aRCMs were cultured for 24 h under various conditions, being low palmitate (LP, basal condition for aRCMs), high palmitate (HP, palmitate/BSA ratio 3:1), and HP supplemented with Resveratrol (R), Quercetin (Q), Berberine (B), Baicalein (Ba), or Isorhamnetin (Iso). After 24 h, cells were lysed and either used for Western blotting or qPCR. (**A**,**B**). Phosphorylation levels of ERK1/2(Thr202/Thr204) in aRCM (*n* = 5) were detected by Western blotting. For quantitative comparison of p-ERK1/2 among the different conditions, these signals were normalized against the respective signal of caveolin-3 protein content (loading control). Representative Western blots of p-ERK1/2 and Cav-3 are displayed. (**C**–**E**). The mRNA levels of *SOD2*, *SIRT3*, and *TNF-α* were detected by qPCR in aRCMs. Transcripts were normalized to actin (*n* = 4). White columns represent basal condition, red columns represent HP condition, blue columns represent prevention of TCM compounds. Bar values are means ± SEM. One-way ANOVA followed by Duncan’s post hoc tests (among the groups, i.e., different culturing conditions) was used for the comparison. * *p* < 0.05 was considered statically significant. (* *p* < 0.05, ** *p* < 0.01, *** *p* < 0.001, **** *p* < 0.0001).

Accumulating evidence reveals that myocardial lipid accumulation is associated with increased oxidative stress due to increased mitochondrial fatty acid oxidation and subsequent mitochondrial dysfunction [10]. As a readout of oxidative stress, ERK1/2 phosphorylation was investigated [35,36]. As expected, HP exposure caused activation of ERK, which was prevented by Quercetin, Berberine, and Isorhamnetin in aRCMs, but not by Resveratrol and Baicalein (Figure 3A) and by all the TCM compounds in HL-1 cells (Supplementary Figure S2A). Mitochondrial superoxide dismutase 2 (SOD2) is also known to have a crucial role in oxidative stress, and activation of SOD2 is accomplished through deacetylation by SIRT3 [37]. As expected, HP exposure caused a decrease in the expression of *SIRT3* and *SOD2* in aRCMs (Figure 3C–E). All TCM compounds not only prevented the reduction in *SIRT3*, but also induced a ~3-fold increase in expression level compared to that of the LP condition (Figure 3D). Furthermore, Berberine and Baicalein, but not Resveratrol, Quercetin, and Isorhamnetin, preserved the expression of *SOD2* (Figure 3C).

Besides oxidative stress, inflammation is a key mechanism involved in the pathogenesis of lipotoxic cardiomyopathy. Previous work has demonstrated inflammation in lipid overload, which features the inflammatory cytokine, *TNF-α* [38]. Incubation with HP for 24 h in aRCMs significantly promoted *TNF-α* expression, which was prevented by Resveratrol, Baicalein, and Isorhamnetin, but not by Quercetin and Berberine (Figure 3E).

### 2.4. TCM Compounds Not Only Prevent but Also Ameliorate Cardiac Contractile Dysfunction in Lipid-Overexposed Cardiomyocytes

**Figure 4:**
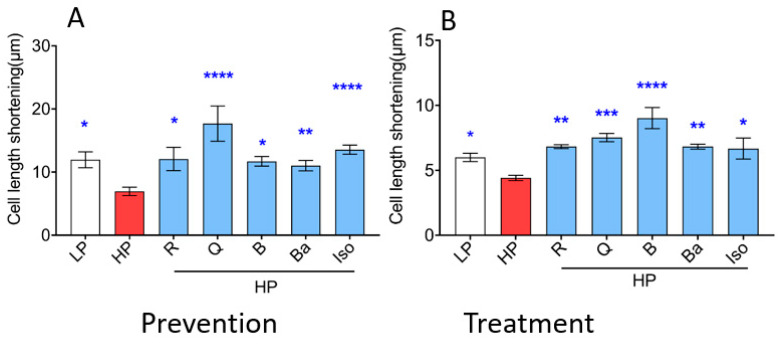
TCM compounds not only prevent but also ameliorate cardiac contractile dysfunction in lipid-overexposed cardiomyocytes. In panel (**A**) (Prevention): aRCM were cultured for 24 h under various conditions, being low palmitate (LP, basal condition for aRCMs), high palmitate (HP, palmitate/BSA ratio 3:1), or HP supplemented with Resveratrol (R), Quercetin (Q), Berberine (B), Baicalein (Ba), or Isorhamnetin (Iso) (*n* = 6). In panel (**B**) (Treatment): aRCMs were incubated for the first 24 h in either low palmitate (LP, basal condition for aRCMs) or high palmitate (HP, palmitate/BSA ratio 3:1). Then, the medium was replaced by either low palmitate (LP, basal condition for aRCMs), high palmitate (HP, palmitate/BSA ratio 3:1), or HP supplemented with Resveratrol (R), Quercetin (Q), Berberine (B), Baicalein (Ba), or Isorhamnetin (Iso) for another 24 h (*n* = 6). Subsequently, contractile properties of aRCMs were assessed at 1 Hz, 30 v, and 5 ms using a video-based cell geometry system to measure sarcomere shortening dynamics during electrostimulation. An example of contraction recordings is in the Supplementary Materials. White columns represent basal condition, red columns represent HP condition, blue columns represent prevention of treatment of TCM compounds Bar values are means ± SEM. One-way ANOVA followed by Duncan’s post hoc tests (among the groups, i.e., different culturing conditions) was used for the comparison. * *p* < 0.05 was considered statically significant. (* *p* < 0.05, ** *p* < 0.01, *** *p* < 0.001, **** *p* < 0.0001).

Lipid overexposure of the heart, as seen in high-fat-diet-fed rats, ultimately leads to contractile dysfunction via increased CD36-mediated myocellular lipid accumulation [39]. Accordingly, in the present study, lipid overexposure-induced contractile dysfunction in aRCM (Figure 4A). Specifically, HP exposure reduced sarcomere shortening by about 40%. Isoproterenol (positive control) increased the sarcomere shortening by 60% (Figure 4A). First, we tested whether the selected TCM compounds were able to prevent the HP-induced loss of contraction upon 24 h co-incubation with HP. Resveratrol, Quercetin, Berberine, Baicalein, and Isorhamnetin increased sarcomere shortening after 24 h co-incubation with HP and, hence, prevented lipid overexposure-induced cardiac contractile dysfunction (Figure 4A). Then, we tested whether the TCM compounds would be able to restore contractile function upon prior establishment of contractile dysfunction. For this, the cardiomyocytes were first incubated with HP for 24 h in the absence of TCM compounds, during which time contractile dysfunction will have developed. Then, for the subsequent 24 h, the TCM compounds were added. It appeared that Resveratrol, Quercetin, Berberine, Baicalein, and Isorhamnetin were capable to restore cardiac contractile dysfunction caused by lipid overexposure in aRCM (Figure 4B).

## 3. Discussion

In the present study, we found that Resveratrol, Quercetin, Berberine, Baicalein, and Isorhamnetin all prevent and ameliorate cardiac contractile dysfunction in lipid-overexposed cardiomyocytes. The common underlying mechanism likely includes the ability of these TCM compounds to prevent lipid accumulation in lipid-overexposed cardiomyocytes. This would be a valid mechanism, given that lipid accumulation is known to be causal for cardiac contractile dysfunction [40]. Below we describe step-by-step the possible shared mechanism(s) by which the different TCM compounds may exert their beneficial effects on the preservation of contraction. 

### 3.1. Linkage between Prevention of Lipid Accumulation by the TCM Compounds and Preservation of Insulin-Stimulated Glucose Uptake

The prevention of lipid accumulation by the TCM compounds was entirely associated with the preservation of insulin-stimulated CD36 translocation. This restoration of trafficking by the TCM compounds implies that CD36 returned to intracellular locations, i.e., storage within endosomes. Otherwise, it would not be available for short-term insulin-stimulated translocation, thereby precluding the cells from taking up excess palmitate [41]. Not only insulin-stimulated CD36 translocation was preserved, but also insulin-stimulated GLUT4 translocation was preserved by the TCM compounds. Importantly, the preservation of insulin-stimulated glucose uptake is known to be downstream of and dependent on the preservation of intracellular CD36 localization within the endosomes and the prevention of lipid accumulation. Namely, upon HP exposure of aRCMs in previous studies, increased CD36 translocation and lipid accumulation preceded the loss of GLUT4 translocation in time. In fact, the time gap between both processes was relatively large [13]. Moreover, a pharmacological blockade of CD36-mediated fatty acid uptake was found to preserve insulin-stimulated glucose uptake in aRCMs exposed to lipid oversupply [42]. In conclusion, the TCM compounds preserve insulin-stimulated GLUT4 translocation/glucose uptake by reducing lipid uptake via the preservation of CD36 traffic (i.e., preferential endosomal storage of CD36).

### 3.2. Mismatch between Preservation of Insulin-Stimulated Glucose Uptake by the TCM Compounds and Impairment of the Canonical Insulin Signaling Pathway

Surprisingly, despite the preservation of insulin-stimulated GLUT4 translocation by all TCM compounds in lipid-overexposed cardiomyocytes, there is inconsistent preservation of the insulin signaling, as determined by the decreased stimulation of AKT phosphorylation by insulin (Figure 2A,B). Then the question rises as to how, by which mechanism, insulin-stimulated GLUT4 translocation is preserved in HP-cultured aRCMs that are treated with Berberine or Baicalein, whereas insulin signaling remains greatly impaired. As part of an explanation, there may be an initial and transient increase in lipid accumulation, which is not detectable anymore after 20 h in the presence of the selected TCM compounds but is still sufficient to induce a more persistent inhibition of insulin signaling. Despite the fact that HP and the TCM compounds were added simultaneously to the cardiomyocytes, this hypothetical transient increase in lipid accumulation may be occurring due to a possible time lag of the TCM compounds to normalize subcellular CD36 distribution upon CD36’s rapid maladaptive lipid-induced translocation to the cell surface that was earlier found to occur within 1 h [13]. The transiently increased lipid species could include DAG and/or ceramides, inhibiting Akt activation via inhibition of upstream insulin signaling or more directly, respectively [41]. The inability of Berberine or Baicalein to preserve insulin signaling and Akt activation has apparently no consequences for their beneficial action on preserving insulin-stimulated GLUT4 translocation/glucose uptake. As earlier established by us [38,43] and also shown with the MK2206 data, insulin-stimulated glucose uptake can normally proceed upon the suppression of Akt activation. In other words, Akt is not rate-limiting for insulin-stimulated glucose uptake. There are certainly also other mechanisms responsible for the lipid-induced loss of glucose uptake, which are more immediately reverted by the restoration of low lipid levels upon their transient rise. These mechanisms may include the competition between lipid droplets and the GLUT4 translocation machinery for the same SNARE proteins, such as SNAP23 [44], so that lowering the lipid droplet content would make SNAP23 re-available for insulin-stimulated GLUT4 translocation. In conclusion, despite the loss of insulin signaling, the TCM compounds preserve insulin-stimulated GLUT4 translocation, which appears to be necessary for the preservation of contractile activity in lipid-overexposed cardiomyocytes. Finally, it is possible that Berberine and Baicalein eventually restore the canonical insulin signaling pathway upon extended incubation times.

### 3.3. Effects of TCM Compounds on Anti-OxidativeAnti-Inflammatory Processes

In LCM, overproduction of reactive oxygen species (ROS) and free radicals occurs, and the antioxidant potential is decreased, inducing oxidative stress [45]. Oxidative stress can aggravate diabetic complications by the activation of pro-inflammatory signals [6,39]. Hence, in general, TCM compounds are considered to display potential effects of treating diabetes and cardiovascular disease due to their strong antioxidant and anti-inflammatory potential. 

Under HP conditions, in the absence of TCM compounds, our findings show an increase in the phosphorylation state of ERK, as well as a downregulation of anti-oxidative/anti-inflammatory defensive mechanisms, such as decreased expression of SIRT3 and SOD2, and upregulation of pro-inflammatory cytokines such as TNF-α (Figure 3). 

While all TCM compounds reduced the occurrence of phosphorylated ERK in HL-1 cells, solely Resveratrol and Baicalein showed a significant decrease in aRCMs. The observed inconsistency may be explained by the different origins and genetic programs of the two cell types (e.g., rat primary cardiomyocyte vs. mouse cancerous cardiomyocyte cell line). 

According to our data, all tested TCM compounds increased SIRT3 expression, but only Baicalein and Berberine also significantly increased SOD2 expression. In line with these findings, Berberine was shown to improve superoxide dismutase and catalase in diabetic mice [46]. Baicalein reportedly provides protection for cellular components against oxidative stress via ROS scavenging, enhances gene expression and antioxidant enzyme activities in diabetic rats [47,48]. Additionally, Quercetin was described as a potent antioxidant by directly scavenging ROS and activating antioxidant enzymes such as SOD in db/db mice [49]. SOD2-mediated ROS reduction is synergistically increased by SIRT3 co-expression, which is cancelled by SIRT3 depletion [50]. Resveratrol inhibited ROS/ERK in diabetic mice [51,52]. Isorhamnetin was reported to reduce lipid peroxidation [33]. Although we can only speculate, Quercetin, Resveratrol, and Isorhamnetin may preferentially target pathways or transcriptional regulators that specifically enhance SIRT3 expression without affecting SOD2 mRNA levels. Anti-oxidative effects of Quercetin, Resveratrol, and Isorhamnetin might then arise, e.g., from post-translational regulation of SOD2 activity or stability, without altering its mRNA abundance. 

The pro-inflammatory cytokine TNF-α plays a crucial role in lipid-overload-induced cardiomyopathy. However, the effects of TCM compounds on TNF-α expression in this setting is not well-known. Evidence from previous work has demonstrated that Quercetin, Resveratrol, Berberine, and Baicalein show great potential in downregulating TNF-α in myocardial ischemia reperfusion injuries in rats [53,54,55,56]. Isorhamnetin displays renoprotective effects in diabetic rats [57]. And, more importantly, our study for the first time indicates that Resveratrol, Baicalein, and Isorhamnetin downregulate TNF-α in lipid-overloaded cardiomyocytes. Quercetin and Berberine failed to show the known effects from ischemia reperfusion injuries in our model of lipotoxic cardiomyopathy, which may be due to different stimuli and experimental settings.

Collectively, in our study, the selected TCM compounds did not have consistent effects on oxidative and inflammatory parameters. Several but not all compounds appeared to suppress ERK signaling, whereas a distinct set of compounds preserved SOD2 expression, and yet again, other compounds prevented TNF-α upregulation. Hence, there is no compound that had a consistent effect on all three oxidative/inflammatory parameters. Given that all selected TCM compounds antagonized CD36-mediated lipid accumulation and preserved insulin-stimulated GLUT4 translocation as well as contractile function, two main conclusions can be drawn: First, the effects of the TCM compounds on oxidative stress/inflammation are independent of the prevention of CD36-mediated lipid accumulation and, hence, have separate beneficial actions. Second, the TCM compounds may exert their beneficial effects on contractile function via the prevention of CD36-mediated lipid accumulation rather than via their anti-oxidative/anti-inflammatory actions.

### 3.4. Limitations

First, although we have clearly demonstrated the protective effect of Resveratrol, Quercetin, Berberine, Baicalein, and Isorhamnetin in isolated cardiomyocytes *in vitro*, the *in vivo* effectiveness has not been tested. Second, our study only superficially demonstrated the regulatory effects of Resveratrol, Quercetin, Berberine, Baicalein, and Isorhamnetin on glucose and lipid metabolism, as well as genes related to inflammation and oxidative stress. We also did not find a specific target or pathway clarifying how individual compound works to produce the observed effects. However, accumulated evidence suggests that compounds derived from Chinese herbal medicine work in a multi-target, multi-pathway manner [58,59].

## 4. Materials and Methods

### 4.1. Compounds

Resveratrol, Quercetin, Berberine, Baicalein, Isorhamnetin, and AKT inhibitor MK2206 were all purchased from Sigma-Aldrich Co (Burlington, MA, USA).

### 4.2. Antibodies

Primary antibodies used in Western blotting analysis are provided in Table S1.

### 4.3. Culturing of HL-1 Cardiomyocytes

The HL-1 cell line was kindly provided by Dr. W. Claycomb (Louisiana State University, New Orleans, LA, USA). HL-1 cells were cultured in Claycomb Medium, supplemented with 1% of penicillin/streptomycin, 0.1 mM norepinephrine, and 2 mM l-glutamine referred as Growth medium. HL-1 cells were seeded in pre-coated plates in Growth medium for 24 h and then changed to control (ctrl) medium which consisted of DMEM-31885 supplemented with 1% penicillin/streptomycin, 0.1 mM norepinephrine, and 1% NEAA. For the high-palmitate (HP) condition, ctrl medium was supplemented for 16 h with 500 μM palmitate and 50 nM insulin (HP) with/without individual TCMs: Resveratrol, 7.5 μM; Quercetin, 0.75 μM; Berberine, 0.1 μM; Baicalein, 0.1 μM; and Isorhamnetin, 0.1 μM (all dissolved in DMSO).

### 4.4. Isolation and Culturing of Primary Rat Cardiomyocytes

Male Lewis rats, weighing 250–340 g, were purchased from Charles River Laboratories (Wilmington, DE, USA) and maintained at the Experimental Animal Facility of Maastricht University. All animals were housed in a controlled environment with a stable temperature of 21–22 °C and a 12:12 h light–dark cycle (light from 7:00–19:00). Animals had free access to food and tap water. All animal procedures were performed in accordance with EU guidelines and national legislation and approved by the Central Committee for Animal Experiments (CCD) and Animal Welfare Body (IVD) of the Maastricht University, Netherlands (AVD 10700202115692). Adult rat cardiomyocytes (aRCM) were isolated using the Langendorff perfusion system, as previously described [43]. All isolated cardiomyocytes were seeded on the laminin-coated plates. After 2 h of adhesion, the culturing medium was changed by either low-palmitate medium (LP, 20 μM palmitate, palmitate/BSA ratios of 0.3:1), high-palmitate (HP, 200 μM palmitate, palmitate/BSA ratios of 3:1), or HP supplemented with individual TCMs: Resveratrol, 7.5 μM; Quercetin, 0.75 μM; Berberine, 0.1 μM; Baicalein, 0.1 μM; and Isorhamnetin, 0.1 μM (all dissolved in DMSO).

### 4.5. Sulforhodamine B (SRB) Colorimetric Assay for Cytotoxicity Screening

After incubation, the culture medium was replaced by a medium containing 5% FCS to minimize background interference. Then, cells were fixed by layering trichloroacetic acid (TCA, final concentration of 10% (*w*/*v*)) and incubated at 4 °C for 1 h. Plates were rinsed under running water, and excess water was flicked from cells. Then, 50 µL of 4% SRB in 1% (*v*/*v*) acetic acid was added to the cells, and they were incubated at room temperature for 30 min. Plates were rapidly de-stained by rinsing in water containing 1% acetic acid (4×) to prevent any leaching of the stain from the cells. The staining of cells in each well was then dissolved in 100 µL of 10 mM non-buffered Tris-base (tris (hydroxymethyl) aminomethane, pH = 10) on a shaker in the dark for 1–4 h. Lastly, plates were read at OD490 nm.

### 4.6. Lipid Droplet Staining: Oil Red O Staining

Lipid droplet staining was measured by Oil red O (ORO) staining. The ORO working solution was prepared by diluting ORO stock solution, which was dissolved in isopropanol at 0.5% in distilled water, in a ratio of 3:2. The ORO working solution was filtered at least twice before use. Cells were cultured on top of coverslips. After culturing, cells were washed with PBS and fixed using 2% formaldehyde for 20 min at 37 °C; then, fixed cells were washed with PBS and incubated with ORO working solution for 10 min. Stained cells were properly washed with PBS and then placed on a glass slide and mounted with a glycerol DABCO mounting solution (10 mL Tris-HCl buffer 0.2 M pF 8.0, 20 mg NaN3, and 2 g DABCO (Sigma-Aldrich Co, Burlington, MA, USA) D2552 1,4-Diazabiclcol[2,2,2]-octane), 90 mL glycerol) to mounting solution. Clear nail polish was used around the coverslips to adhere them in place. Images were acquired with a brightfield microscope (Axiophot, Zeiss, Oberkochen, Germany) with a 20×, NA: 0.5 dry objective. Images were analyzed with Fiji-win 64 [60,61].

### 4.7. Surface-Protein Biotinylation for Detecting GLUT4 and CD36 Translocation

Surface-protein biotinylation was measured as previously described [62]. Briefly, after 24 h culture under various conditions, aRCMs were incubated for 30 min with (or without) 100 nM insulin. During the last 10 min of this period, the cell-impermeable reagent Sulfo NHS-LC-biotin (Thermo Fisher Scientific, Fremont, CA, USA) was added. Thereafter, the cells were washed and lysed for subsequent IP with streptavidin beads (Thermo Fisher Scientific, Fremont, CA, USA). Upon further washing and elution of the biotinylated proteins from the beads, samples that contained the biotinylated proteins were used for Western blotting analysis of CD36 and insulin-regulated aminopeptidase (IRAP, reflecting GLUT4 trafficking).

### 4.8. Determination of Insulin Sensitivity

After culturing, aRCMs and HL-1 cells were exposed to insulin (aRCMs: 100 nM; HL-1 cells: 200 nM) for short-term treatment of 30 min to compare basal phosphorylation to insulin-stimulated phosphorylation of AKT. Thereafter, all treated cells were directly lysed in sample buffer, and the expression levels of phospho-Ser473-Akt (pAKT) and phospho-Ser235/236-S6 (pS6) were detected by SDS-polyacrylamide gel electrophoresis, followed by Western blotting.

### 4.9. Measurement of Glucose Uptake

[^3^H] deoxyglucose uptake into HL-1 cells and aRCMs were assessed as previously described [63]. Briefly, cells were seeded on pre-coated plates. Following stimulation, [^3^H] deoxyglucose is taken up into the cell without being oxidized. Subsequently, accumulated [^3^H] deoxyglucose was quantified using a β-counter.

### 4.10. Real-Time RT-PCR

Total RNA was extracted from heart tissue by use of Trizol reagent (Sigma, Burlington, MA, USA) and reverse-transcribed by use of a cDNA reverse-transcription kit (qScript cDNA Synthesis Kit). The sequences of primers are listed in Table S2. The mRNA levels were calculated on the basis of threshold cycle (CT) values. The mRNA expression of target genes was normalized to that of β-actin as the control by the 2^−ΔΔCT^ method. Each experiment was repeated in triplicate.

### 4.11. Measurement of Cardiomyocyte Contraction Dynamics

Contractile properties of aRCMs were assessed at 1 Hz, 30 v, and 5 ms using a video-based cell geometry system to measure sarcomere shortening dynamics (IonOptix, Milton, MA, USA). The digitized recordings acquired with the camera were converted to AVI format using Virtual dub2 (https://sourceforge.net/projects/vdfiltermod/, accessed on 12 October 2022) and then analyzed with Fiji and the Beats analysis tool created by our lab (https://martijnhoes.shinyapps.io/myomate/, accessed on 12 October 2022).

### 4.12. Statistics

Statistical analyses were performed using IBM SPSS Statistics 23 (SPSS Inc., Chicago, IL, USA) and GraphPad 9.0 PRISM. All data were tested for normal distribution. Briefly, all data were compared using a one-way ANOVA followed by Duncan’s post hoc tests (among the groups, i.e., different culturing conditions), or paired Student’s *t*-test (within groups, i.e., when analyzing short-term insulin effect). All data are presented as mean ± SEM. *p*-values < 0.05 are considered statistically significant. (* *p* < 0.05, ** *p* < 0.01, *** *p* < 0.001, **** *p* < 0.0001)

## 5. Conclusions

In summary, Resveratrol, Quercetin, Berberine, Baicalein, and Isorhamnetin were found to increase insulin-stimulated glucose uptake and decrease lipid accumulation, thereby ameliorating lipotoxicity in lipid-overexposed cardiomyocytes. Most importantly, all five compounds prevented and restored lipid-induced contractile dysfunction. These findings are in line with those of previous studies, demonstrating that these compounds have antidiabetic potential in hepatocytes, skeletal muscle cells, and adipocytes. The current study, therefore, adds a reference for further clinical testing and the construction of possible treatment guidelines for LCM.

## Data Availability

The datasets used and/or analyzed during the current study are available from the corresponding author upon reasonable request.

## Supplementary Materials

The following are available online at https://www.mdpi.com/article/10.3390/ijms25158131/s1.

## Abbreviations

TCM	Traditional Chinese Medicine
LCM	lipotoxic cardiomyopathy
aRCM	adult rat cardiomyocytes
R	Resveratrol
Q	Quercetin
B	Berberine
Ba	Baicalein
Iso	Isorhamnetin
HP	high palmitate
LP	low palmitate
IRAP	insulin-regulated aminopeptidase
LCFA	long-chain fatty acids
ORO	Oil red O
SRB	Sulforhodamine B
