# Supplementary material for: Specific Compounds Derived from Traditional Chinese Medicine Ameliorate Lipid-Induced Contractile Dysfunction in Cardiomyocytes
Source: Int J Mol Sci. 2024 Jul 25;25(15):8131. doi: 10.3390/ijms25158131 (PMC11311577; doi:10.3390/ijms25158131)
Supplement: Supplementary file 1 [file ijms-25-08131-s001.zip › ijms-3040020-supplementary.pdf]

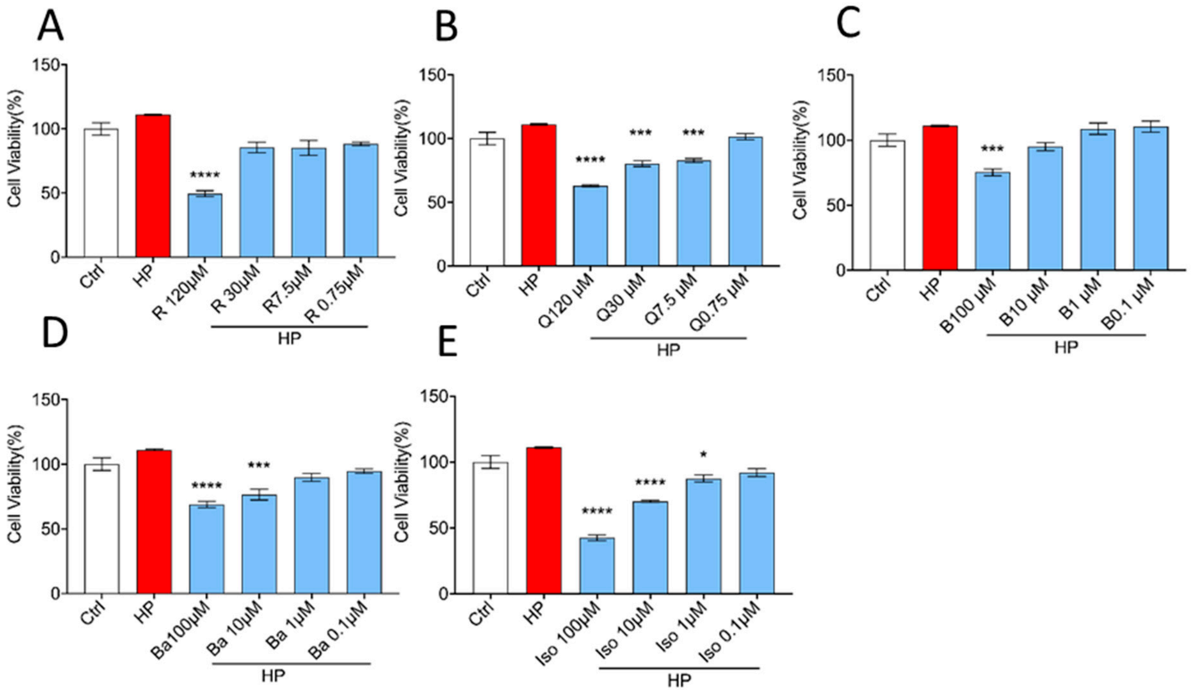

Supplemental Figure S1. Effects of individual TCM compounds on cell viability in aRCMs. Cells were incubated for 24 h under different conditions: low palmitate, (LP, basal condition for aRCMs; palmitate/BSA ratio 0.3:1), high palmitate (HP, palmitate/BSA ratio 3:1), HP supplemented with Resveratrol (A), Quercetin (B), Berberine (C), Baicalein (D), or Isorhamnetin (E) at decreasing concentrations. Subsequently, cells were subjected to the SRB assay (n = 4). White columns represent basal condition, red columns represent HP condition, blue columns rep-resent prevention of TCM compounds. Data are presented as means ± SEM. One-way ANOVA followed by Duncan's post hoc tests (among the groups, i.e., different culturing conditions), or paired Student's t-test (within groups, i.e., when analyzing short-term insulin effect) was used for the comparison. *p < 0.05 was considered statically significant. ((*p < 0.05, ***p < 0.001, ****p < 0.0001))

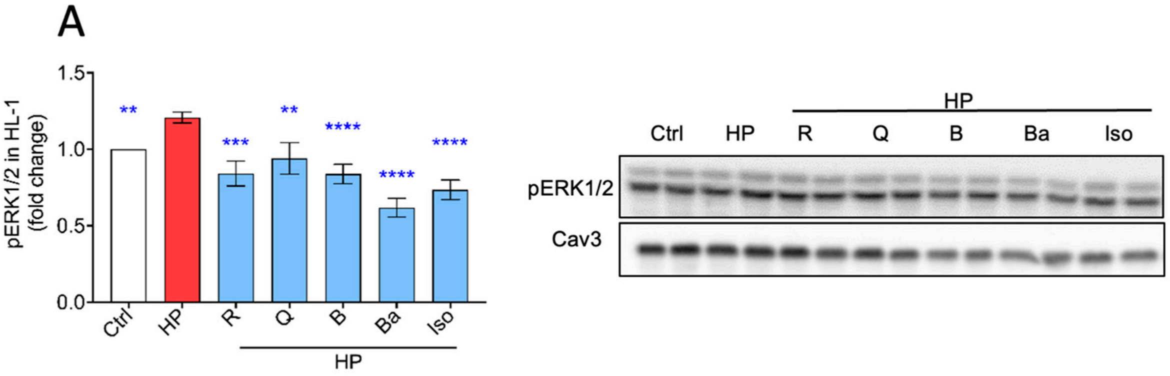

Supplemental Figure S2 (related to Figure 3A-B). Effects of individual TCM compounds on phosphorylation levels of ERK1/2(Thr202/Thr204) in HL-1 cells (n=5) was determined by western blotting. Cells were incubated for 24 h under different conditions: low palmitate, (LP, basal condition for aRCMs; palmitate/BSA ratio 0.3:1), high palmitate (HP, palmitate/BSA ratio 3:1), HP supplemented with Resveratrol (R), Quercetin (Q), Berberine (B), Baicalein (Ba), or Isorhamnetin (Iso). A: For quantitative comparison of p-ERK1/2 among the different conditions, these signals were normalized against the respective signal of caveolin-3 protein content (loading control). B: Representative western blots of p-ERK1/2 and Cav-3 are displayed. White columns represent basal condition, red columns represent HP condition, blue columns rep-resent prevention of TCM compounds.Data are presented as means ± SEM. One-way ANOVA followed by Duncan's post hoc tests (among the groups, i.e., different culturing conditions), or paired Student's t-test (within groups, i.e., when analyzing short-term insulin effect) was used for the comparison. *p < 0.05 was considered statically significant. (**p < 0.01, ***p < 0.001, ****p < 0.0001)

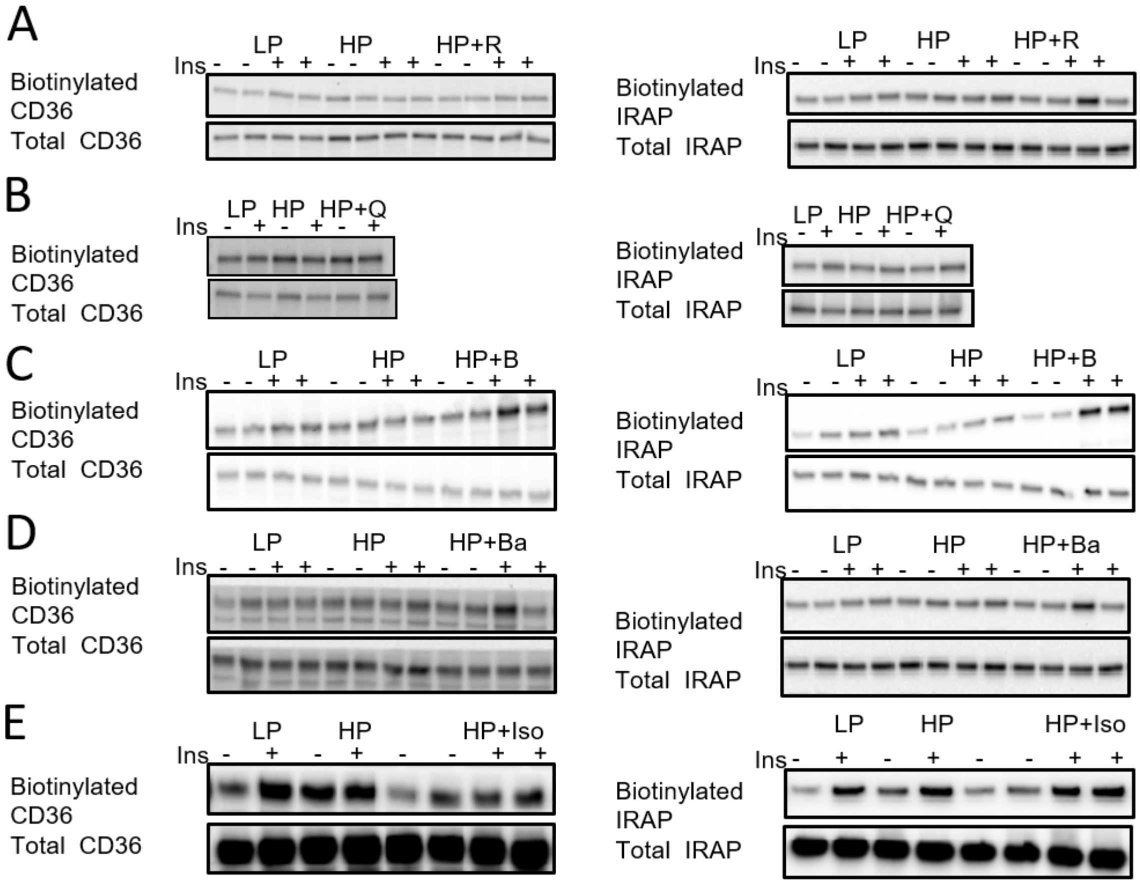

Supplemental Figure S3 (related to Figure 1C-G, Figure 2E-I). Effects of individual TCM compounds on cell surface levels of CD36 and GLUT4 were determined by western blotting, R (n = 4), Q (n = 5), B (n = 4), Ba (n = 5), Iso (n = 4). In panels A-E, cells were cultured for 24 h under various conditions, being low palmitate (LP, basal condition for aRCMs), high palmitate (HP, palmitate/BSA ratio 3:1), HP supplemented with Resveratrol (R), Quercetin (Q), Berberine (B), Baicalein (Ba), or Isorhamnetin (Iso). After 24 h, cells were short-term (30 min) incubated without/with insulin (aRCMs: 100 nM insulin). And then, cells were subjected to biotinylation assay. Representative western blot of insulin-regulated CD36 and aminopeptidase (IRAP, which reflects GLUT4 translocation) are shown in biotin- immunoprecipitation and total lysate fraction. Left panel were biotin-immunoprecipitation and total lysate fraction for CD36, right panel were biotin-immunoprecipitation and total lysate fraction for GLUT4.

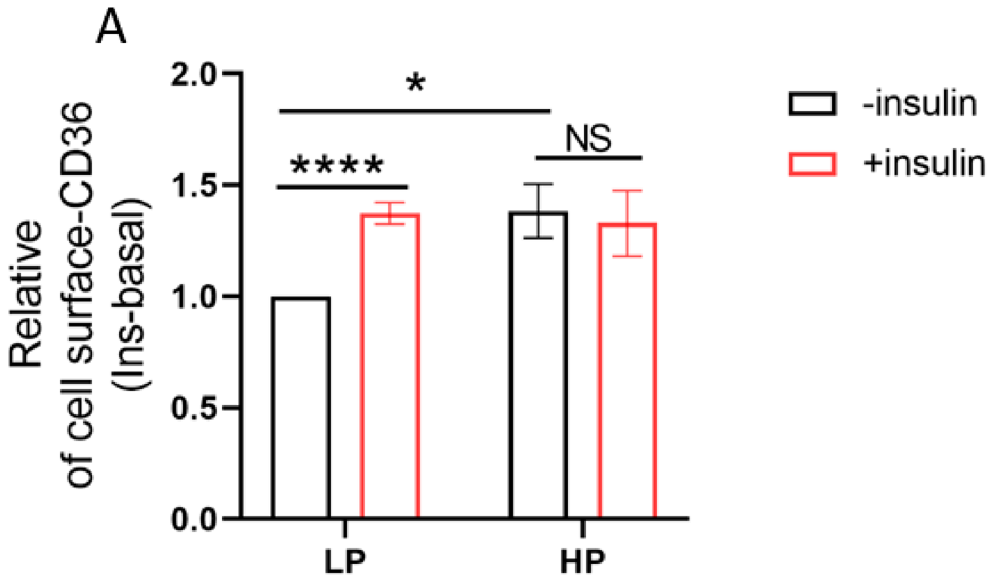

Supplemental Figure S4 (related to Figure 1C-G). A, assessment of cell surface CD36 in aRCMs between LP and HP. cells were incubated for 24 h under different conditions, being low palmitate, (LP, basal condition for aRCMs; palmitate/BSA ratio 0.3:1) and high palmitate (HP, palmitate/BSA ratio 3:1), After 24 h, cells were short-term (30 min) incubated without/with 100 nM insulin. Then, cells were subjected to the Biotinylation assay. CD36 was assessed using Western blotting in both biotin immunoprecipitations and total cell lysates, followed by quantification combined all experiments ($n = 22$). White columns represent basal condition, red represent insulin stimulation condition. Data are presented as mean ± SEM. One-way ANOVA followed by Duncan's post hoc tests (among the groups, i.e., different culturing conditions) was used for the comparison. *NS* represents non significant,*$p < 0.05$ was considered statically significant. (*$p < 0.05$, ****$p < 0.0001$)

Supplement 5. Example of contraction recording

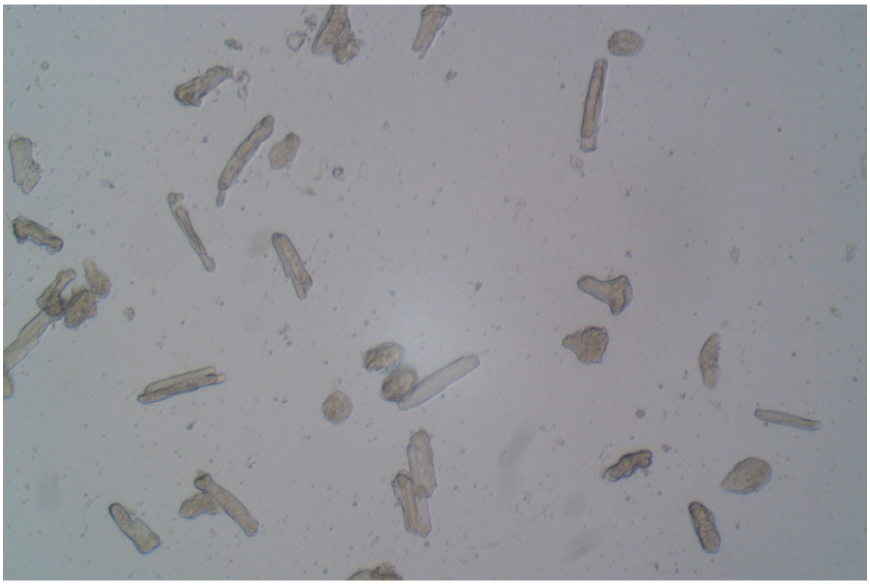

Supplement Figure S5 (related to Figure 4A-B). Assessment of contractile properties of aRCMs. aRCMs were cultured for 24 h under various conditions, being low palmitate (LP, basal condition for aRCMs), high palmitate (HP, palmitate/BSA ratio 3:1), HP supplemented with Resveratrol (R), Quercetin (Q), Berberine (B), Baicalein

(Ba), or Isorhamnetin (Iso), (n = 6). In panel B. (Treatment); aRCMs were incubated for the first 24 h in either low palmitate (LP, basal condition for aRCMs) LP or high palmitate (HP, palmitate/BSA ratio 3:1) HP. Then, the medium was replaced by either low palmitate (LP, basal condition for aRCMs), high palmitate (HP, palmitate/BSA ratio 3:1), HP supplemented with Resveratrol (R), Quercetin (Q), Berberine (B), Baicalein (Ba), or Isorhamnetin (Iso) LP, HP, or HP supplemented with Resveratrol (R), Quercetin (Q), Berberine (B), Baicalein (Ba), or Isorhamnetin (Iso) for another 24 h, (n = 6).
Subsequently, contractile properties of aRCMs were assessed at 1Hz, 30v, and 5ms using a video-based cell geometry system to measure sarcomere shortening dynamics during electrostimulation. Example of contraction recordings were shown.

Table S1. Antibodies used in this study

Antibodies	Sorce	Identifier
p-AKT(Ser473)	Cell signaling	#9271
Total-AKT	Cell signaling	#9272
Insulin-regulated aminopeptidase (IRAP)	Cell signaling	#MABN483
CD36(MO25)	Gift from Dr.N.Tandon	NO
GLUT4	Millipore	#07-1404
Caveolin-3	BD transduction Laboratories	#610421
p-ERK1/2	Cell signaling	#9101
Total ERK	Cell signaling	#9271

Table S2. Primers used in this study.

Primers		Sequences
SIRT3	F	5‘-CATCGACGGGCTTGAGAGAG
	R	5’-CAAAAGGCTCCACCCGTATGT
TNF-a	F	5‘-ATGGGCTCCCTCTCATCAGT
	R	5’-GCTTGGTGGTTTGCTACGAC
SOD2	F	5‘-AGGAGCAAGGTCGCTTACAG
	R	5’-CGTGCTCCCACACATCAATC
b-Actin	F	5'- CTAAGGCCAACCGTGAAAAG
	R	5'- AACACAGCCTGGATGGCTAC
